# Sampling strategies for species with high breeding-site fidelity: A case study in burrow-nesting seabirds

**DOI:** 10.1371/journal.pone.0221625

**Published:** 2019-08-27

**Authors:** Gavin E. Arneill, Christopher M. Perrins, Matt J. Wood, David Murphy, Luca Pisani, Mark J. Jessopp, John L. Quinn

**Affiliations:** 1 School of Biological, Earth and Environmental Sciences, University College Cork, Cork, Ireland; 2 MaREI Centre, Environmental Research Institute, University College Cork, Ringaskiddy, Ireland; 3 Edward Grey Institute of Field Ornithology, Department of Zoology, University of Oxford, Oxford, United Kingdom; 4 School of Natural and Social Sciences, University of Gloucestershire, Cheltenham, United Kingdom; University of Reunion Island, RÉUNION

## Abstract

Sampling approaches used to census and monitor populations of flora and fauna are diverse, ranging from simple random sampling to complex hierarchal stratified designs. Usually the approach taken is determined by the spatial and temporal distribution of the study population, along with other characteristics of the focal species. Long-term monitoring programs used to assess seabird population trends are facilitated by their high site fidelity, but are often hampered by large and difficult to access colonies, with highly variable densities that require intensive survey. We aimed to determine the sampling effort required to (a) estimate population size with a high degree of confidence, and (b) detect different scenarios of population change in a regionally important species in the Atlantic, the Manx shearwater (*Puffinus puffinus*). Analyses were carried out using data collected from tape-playback surveys on four islands in the North Atlantic. To explore how sampling effort influenced confidence around abundance estimates, we used the heuristic approach of imagining the areas sampled represented the total population, and bootstrapped varying proportions of subsamples. This revealed that abundance estimates vary dramatically when less than half of all plots (*n* dependent on the size of the site) is randomly subsampled, leading to an unacceptable lack of confidence in population estimates. Confidence is substantially improved using a multi-stage stratified approach based on previous information on distribution in the colonies. In reality, this could lead to reducing the number of plots required by up to 80%. Furthermore, power analyses suggested that random selection of monitoring plots using a matched pairs approach generates little power to detect overall population changes of 10%, and density-dependent changes as large as 50%, because variation in density between plots is so high. Current monitoring programs have a high probability of failing to detect population-level changes due to inappropriate sampling efforts. Focusing sampling in areas of high density with low plot to plot variance dramatically increases the power to detect year to year population change, albeit at the risk of not detecting increases in low density areas, which may be an unavoidable strategy when resources are limited. We discuss how challenging populations with similar features to seabirds might be censused and monitored most effectively.

## Introduction

The need for robust population census and monitoring becomes ever more pressing as anthropogenic impacts intensify [1–3]. Much recent research aims to improve census and monitoring practices by modifying existing methods [4,5] and utilising technological advances—for example drones and automated acoustic recording devices [6–8]—with the aim of reducing costs [6,9]. Nevertheless, basic sampling techniques that underpin these approaches remain to be refined and standardised, not least because some population size estimates and trends are questionable due to inconsistencies and errors in the methods employed [10–12].

Simple random sampling is often carried out in field studies to estimate densities and monitor populations, especially when species are highly mobile [10,11]. However, these methods may be unsuitable, or indeed unnecessary, when sampling in logistically challenging areas and where individual organisms are static or show high site fidelity [12,13]. Moreover, many species distributions are highly clustered, and random sampling necessitates a uniform distribution for small samples to be truly representative [14,15]. If spatial patterns of distribution are known *a priori*, this information can be used to obtain more accurate estimates by stratifying sampling approaches. In ecology, stratification is typically carried out using strata across geographical space, most often defined by distinct habitat types [16,17], and abundance estimates for each strata are then combined to give an overall estimate for the area. This approach has been successful in census and monitoring efforts across many taxa [18,19]. Further complexity can be added in the form of multi-stage stratification, often used in pharmaceutical and educational research [20,21]. In this hierarchical design samples are drawn and then subdivided based on another known variable, e.g., density or habitat. Multi-stage stratification is not commonly used in ecological research yet is applicable in certain instances, such as in repeat censuses where baseline distribution data is available to reduce the effect of variation between strata. The sampling approach used will therefore determine the population estimates and surrounding confidence interval attained from any effort.

Generating baseline population estimates at a given time is a crucial aim in conservation but monitoring these populations over time is equally important. In some groups, such as wading bird species or cliff and ground nesting seabirds, whole population counts of individuals are possible [22,23]. For many species, sampling is more appropriate [24,25], which is typically done by sampling population densities in a number of fixed sample plots regularly over time [26–29]. These sampling approaches are likely to be suitable when distributions do not change rapidly over time [12], and when species are patchily distributed for example within a specific habitat type, precluding the use of random sampling. One group where this is largely thought to be true is in seabirds, which show high nest-site fidelity, are patchy in their distribution, and are migratory, thus only accessible during their breeding season when they return to land.

As apex predators that feed on prey from a range of trophic levels, seabirds are not only key qualitative indicators of the world’s largest biome, they are also among the most threatened vertebrates in the world [3,30,31]. Global monitoring has shown that, although some are increasing, many seabird populations are in decline [3,32–34]. This is primarily because of their sensitivity to invasive mammals, overfishing, by-catch, marine pollution, disturbance, habitat destruction, and climate change [3]. However, there remains considerable uncertainty over the status and trends across all seabird species because most studies are biased towards species that are easy to observe nesting on cliffs or on the ground where whole-colony counts are often possible [35–38]. Burrow-nesting seabird species are amongst the most threatened of all seabirds [39,40], and yet detailed population monitoring studies are rare. For example, in Paleczny *et al*.’s review [32], approximately 46% of the species not considered (*n* = 162) were burrow-nesters. The main reason burrow-nesting species are poorly represented is that they are extremely difficult to census. Many Procellariiformes, for example, are remarkably difficult to census not just because they nest underground, but also because they coexist with other burrowing species and only return to breeding colonies at night [41].

The Manx shearwater (*Puffinus puffinus*) breeds across the North Atlantic, with over 90% of the global population on offshore islands around Britain and Ireland [42]. Thorough monitoring efforts for this species commenced with the development of the tape-playback method by James and Robertson [43], since used in several censuses [41,44]. Nevertheless, there is considerable uncertainty over population size and trends, notably because it remains unclear how to sample individual colonies effectively [45,46]. Here, we assess the performance of different sampling strategies across multiple colonies, using data collected from tape-playback surveys, and a bootstrapping approach to determine the levels of variation associated with different subsampling efforts. Subsampling the sampled area allows inference to be made on real data capturing the spatial variation within colonies, rather than simulated or extrapolated abundance estimates. We test the efficiency of a cluster sampling and a multi-stage stratified sampling approach. Cluster sampling separates plots based on the presence or absence of breeding burrows from initial baseline surveys, and subsequent sampling is only carried out within areas containing at least one breeding burrow. In multi-stage stratification, the randomly selected plots are stratified by different densities, and sampling occurs within each stratum.

We then examine the statistical power to detect simulated population changes across two censuses by subsampling variable numbers of plots, which we did for three different scenarios of population change. Note that the statistical power reported in each scenario reflects change in the population, irrespective of whether such change is an increase or decrease in the population, as the effect size (Cohen’s *d* [47]) that determines statistical power remains the same in both scenarios. In the first instance, our study aimed to inform the design of sampling strategies for obtaining abundance estimates and detecting population changes across national scales. However, it can also inform monitoring trends across any taxa, avian or otherwise, with similar life history and ecological characteristics.

## Methods

### Tape-playback surveys

**Fig 1:**
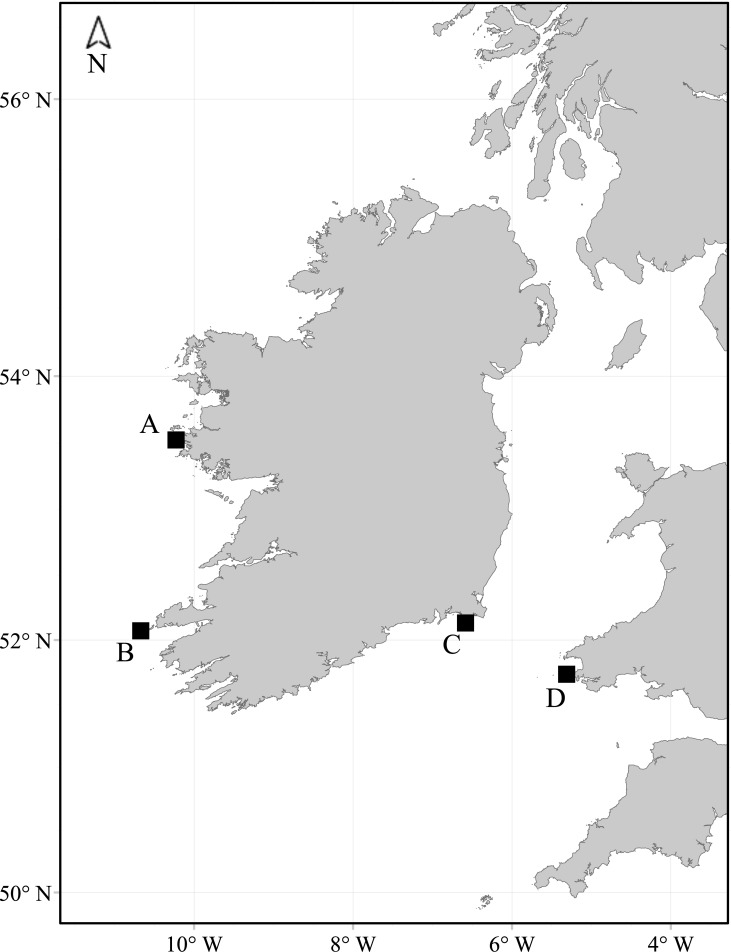
Distribution of Manx shearwater study colonies indicated on the map: (A) High Island, Co. Galway, (B) Inishvickillane, Co. Kerry, (C) Little Saltee, Co. Wexford and (D) Skomer, Pembrokeshire, Wales.

Tape-playback surveys were used to census Skomer, Wales (2011), and three islands in Ireland; Little Saltee (2013), High Island (2015) and Inishvickillane (2016) (Fig 1). Surveys were carried out within the incubation and early chick-rearing periods as the likelihood of a breeding adult being present in the burrow drops sharply once the chick hatches. Tape-playback methods used in censusing burrow-nesting seabirds aim to evoke a behavioural response from a breeding bird, and if a response is received the burrow is thought to host a breeding pair and is defined as an apparently occupied burrow (AOB). Playbacks were conducted during the day to minimise the inclusion of non-breeding birds in burrows [42] and played at burrow entrances at a natural volume (ca. 55dB) for three to four call cycles (approximately 15 seconds) or less if an immediate response was received [41]. Male Manx shearwater calls were used as they are known to elicit a higher number of responses compared to female calls [5,48]. The recordings used for playback surveys in Ireland were from birds recorded on the Pembrokeshire Islands in Wales, as foreign calls are known to elicit a higher response rate in other Procellariiformes [49]. This differed on Skomer where the calls used were of birds from the neighbouring island, Skokholm. Differences in the calls used during the respective survey efforts have no effect on the analyses here, as colony-specific response rates were calculated and applied at the site level. Across all surveys, sample plots were delineated using ArcGIS (ESRI, versions 10–10.2.2) and the order in which plots were surveyed was randomised to reduce any potential temporal bias.

Previous studies using tape-playback methods to census burrow-nesting seabirds applied a correction factor of 1.98 to the total number of responses received, correcting for the fact that occupied burrows respond approximately 50% of the time [45]. However, further work has shown that response rates to tape-playbacks not only vary across species, but spatially and temporally within a breeding population [5,49], advocating the calculation and application of local response rates during these surveys. Here, we calculated colony-specific response rates for High Island, Inishvickillane and Skomer by visiting burrows that were known to be occupied multiple times (30 AOBs on High Island, 4 times; 76 AOBs on Inishvickillane, 9 times; 33 AOBs on Skomer, 8 times). Specifically, tape-playbacks were carried out across the burrows of known occupancy to determine the proportion of responses received. This was repeated at least four times on each of the three sites to calculate the mean and variation in response rates to be calculated across these trials. To minimize pseudo-replication [50] in the repeated tape-playbacks necessary to obtain response rates, recordings from multiple individuals were used in a random order. Trials were separated by at least 24 hours to reduce playback habituation and we assumed that response rate did not change with time of day [5]. No local response rate was calculated for Little Saltee, the average response rate from other Irish colonies recorded during Seabird 2000 was used in its place [42].

### Abundance estimates

**Fig 2:**
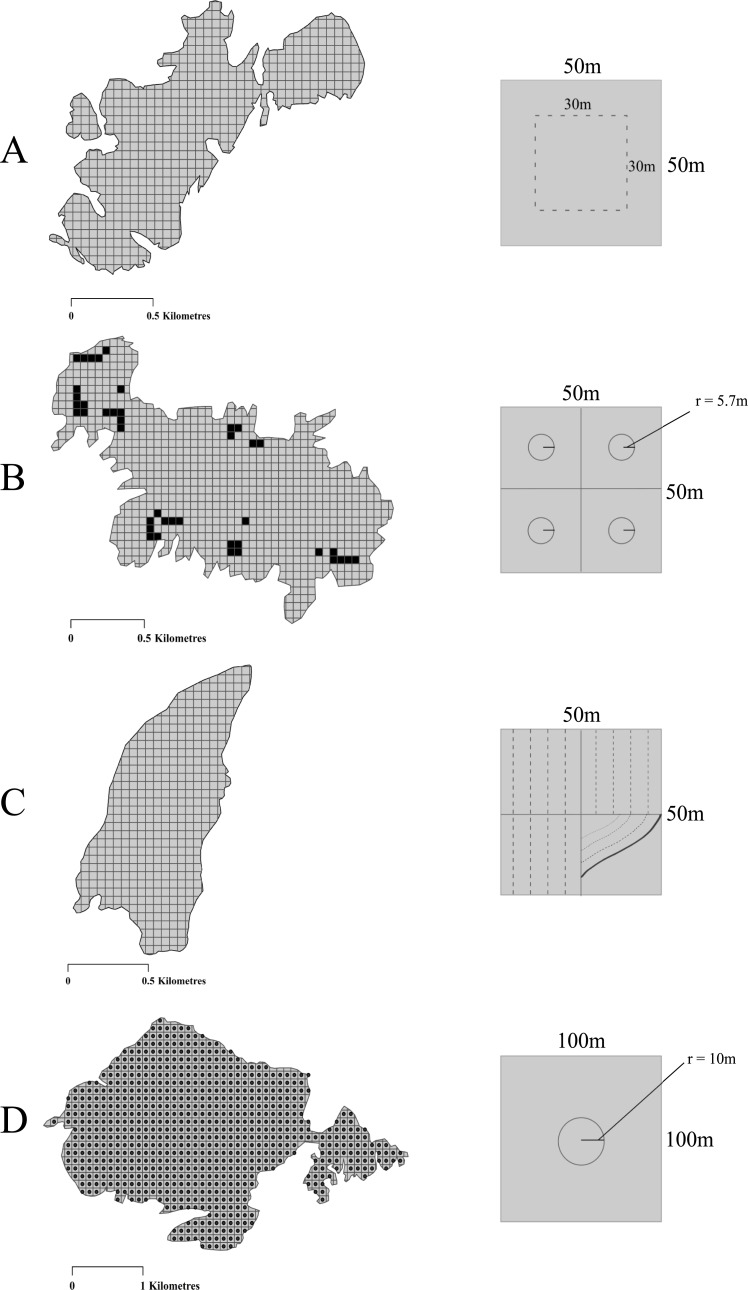
**Schematic outlining the sampling strategies used during playback surveys of (A) High Island, Co. Galway, (B) Inishvickillane, Co. Kerry, (C) Little Saltee, Co. Wexford and (D) Skomer Island, Pembrokeshire, Wales**. The black squares in B indicate the 50x50m plots that had at least one burrow present.

We used census data available from four study colonies where different sampling strategies had been used; therefore, the posthoc analyses were carried out on each island separately. Abundance estimates were generated using a combination of whole-island counts (Little Saltee) and sampling using either a random sampling approach (High Island, Skomer [44]), or a clustered approach (Inishvickillane) based on the presence or absence of at least one AOB. Time constraints associated with access to Inishvickillane warranted the clustered design, whereas on High Island sampling the entire island was possible. The survey on Little Saltee covered 100% of the workable area on the island, while approximately 38%, 16% and 3.5% was sampled on High Island, Inishvickillane and Skomer respectively. Sample plots on High Island were 30m x 30m within each 50m x 50m grid square. To determine the distribution of burrows across Inishvickillane, transects were carried out in a north-south direction through the centre point of each grid square (50m apart) across the entire island. These initial transects did not involve carrying out tape-playbacks at individual burrow entrances and solely noted the presence or absence of burrows within each plot, thus were not time-consuming. This presence/absence data was then used to design the clustered sampling approach, which involved the use of circular sampling plots with a radius of 5.7m within 25m x 25m plots that contained burrows (Fig 2A and 2B). On Little Saltee, rectangular plots (50m x 10m) were used to survey inland areas, while plots next to the coast used a belted transect (10m width) approach to follow the coastline (Fig 2(C)). The combination of the two approaches on Little Saltee allowed whole-island coverage that we are confident incorporated all of the breeding population. On Skomer, tape-playbacks were carried out using circular sampling plots with a radius of 10m in the centre point, or as near as safely possible, of predefined 100m x 100m grid squares across the island (Fig 2D; see Perrins et al. [44]).

### Subsampling approaches

In this heuristic exercise, the total area sampled for each island is treated as a population; thus, in reality the abundance estimates reported here are for the area sampled on each island, not extrapolated estimates for the entire island which are reported elsewhere [44,51]. Given that the plots were sampled randomly, we assume that the variability in the density of plots selected were representative of the variability across all plots in each entire population. Thus, the estimates of confidence in abundance and the power to detect change should be the same as for the entire population. It is important to note that although a clustered design was used on Inishvickillane, random bootstrap sampling of the randomly sampled smaller plots within the larger 50x50m grid squares was still possible as not all of these plots contained breeding burrows. To illustrate the variation in subsampling efforts using random, clustered and multi-stage stratified (‘stratified’) approaches, bootstrap analyses were carried out using the statistical software ‘R’ version 3.3.2. Random sampling involved subsampling from all plots within a site. In the clustered approach, indicative of sampling when presence or absence is known in an area, subsamples were taken only from sampled plots in which at least one AOB was found. In the stratified approach, which is relevant where repeat census efforts are conducted with a known baseline breeding distribution *a priori*, the plots were stratified for four quantiles (0–25%, 25–50%, 50–75% and 75–100%) of plot density, and proportionately subsampled within each stratum. Bootstrap resampling was carried out in 10% increments from 10% to 100% of all plots; thus for these approaches, 10% is 10% of the total sampled plots, not 10% of the entire island’s area. Resampling was repeated 10,000 times; the means of all bootstrapped subsamples approximate the actual abundance due to the large number of iterations. To incorporate the uncertainty around the calculated response rates, bootstrapping was repeated across the range of calculated response rates and abundance estimates were combined. Levene’s test for equality in variances was used across all bootstrapped samples to compare across sampling approaches.

### Detecting population change

Power analyses were used to assess how effective subsampling plots would be at detecting different simulated changes in population density across two independent censuses. This was carried out using three different simulations: (1) where there was a change across the entire colony, plot-specific changes were applied in a normal distribution centred around a 10, 20, 30, 40 and 50% overall population change and the monitoring plots are selected at random; (2) where there was a change only in the high-density areas and the monitoring plots are selected at random, simulating for example, the destruction of favourable habitat or the introduction of a disease with density dependent transmission (e.g.[52]). In simulation 2, the top 25% densest plots were subject to normally distributed simulated change, producing overall population difference in increments of 10% up to 50%. In simulation (3), changes were simulated in a normal distribution across all plots, and the selection of monitoring plots was restricted to the areas of highest density (top 25%) in a clustered approach. Many existing monitoring programmes of burrow-nesting species sample less than 50 plots [53,54]; thus, we calculated the statistical power associated with sampling 10–50 plots, in increments of 10. To show the statistical power associated with the different simulated changes and subsampling efforts, the packages “effsize” and “pwr” were used in the statistical software ‘R’ (version 3.3.2). The package “pwr” uses Cohen’s *d* effect size [47] that was calculated for the simulated changes in “effsize” in a match-pair design. The 95% confidence intervals of statistical power are reported here to demonstrate the precision of the power associated with each simulation.

## Results

### Abundance estimates and bootstrapping

**Fig 3:**
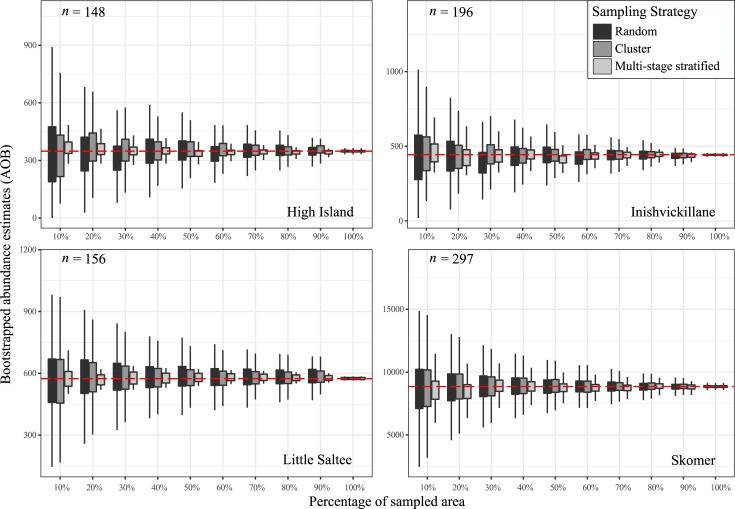
Bootstrapped abundance estimates of the sampled area on four Manx shearwater colonies in Ireland and Wales. **Boxplots show the range of bootstrapped abundance estimates associated with random sampling, clustered sampling, and multi-stage stratified sampling in plots of different densities defined by the quartiles.** The boxes contain the middle 50% of abundance estimates while the whiskers contain the upper and lower 25% of the abundance estimates. The broken red line represents the actual abundance of the entire area sampled (not equating to the entire colony). The total number of plots sampled on each island is denoted with “n”.

A total of 5,183 responses were elicited from playbacks on 21,756 burrows across all study sites over the four censuses. The number of responses and playbacks conducted on High Island, Inishvickillane, Little Saltee and Skomer were: 176/1,599; 224/1,254; 308/5040 and 4,475/13,863 respectively. Local response rates were calculated at 0.55 ± SE 0.07, 0.49 ± SE 0.03 and 0.403 ± SE 0.025 on High Island, Inishvickillane and Skomer respectively. The actual abundance estimates for the area sampled on each island are represented by the broken red line in Fig 3.

Abundance estimates obtained from the bootstrapping analyses across different sampling efforts for the different sampling approaches are shown in Fig 3. As expected, increasing sampling area led to narrower ranges in the bootstrapped abundance estimates for all study sites (Fig 3). Levene’s test for equality in variances showed a clear advantage of clustered and stratified sampling approaches over random sampling; results for each comparison are outlined in Table 1. Comparing random and clustered sampling approaches, all comparisons for both Little Saltee and High Island produced statistically significant (*p* <0.001) differences, with less variation in the estimated abundances when using clustered approaches. A similar result was found for Inishvickillane apart from one comparison at 30% sampling. Skomer had fewer significant differences, with two sampling levels showing no statistically significant (*p* >0.05) difference in variance (see Fig 3 and Table 1(i)). Comparing random and stratified approaches showed that all comparisons across all sites revealed a significant (*p* <0.001) reduction in variance (see Table 1(ii)). Thus, the stratified approach proved the most effective at reducing the variance in bootstrapped estimates.

**Table 1:** Levene’s test comparing the variance in the range of bootstrapped abundance estimates of Manx shearwaters breeding on four islands off of Ireland and Wales between (i) random and clustered sampling approaches and (ii) random and stratified sampling approaches. Both the F-statistic and *p* value are reported here, outlining the significance of the differences between the variances in the abundance estimates. The significant difference corresponds to lower variability in clustered and stratified approaches compared to random sampling.

% Area Sampled	High Island		Inishvickillane		Little Saltee		Skomer	
(i) *Random vs*. *Clustered*								
	*F*	*p*	*F*	*p*	*F*	*p*	*F*	*p*
10%	84.3	**<0.001**	47.6	**<0.001**	35.5	**<0.001**	3.9	**0.047**
20%	42.9	**<0.001**	65.1	**<0.001**	106.7	**<0.001**	2.9	0.083
30%	17.4	**<0.001**	7.6	**0.001**	132.9	**<0.001**	8.8	**0.003**
40%	82.8	**<0.001**	63.7	**<0.001**	86.9	**<0.001**	5.9	**0.01**
50%	65.9	**<0.001**	74	**<0.001**	157.4	**<0.001**	4.3	**0.038**
60%	35.5	**<0.001**	35.6	**<0.001**	154.3	**<0.001**	1.1	0.3
70%	85.9	**<0.001**	22.6	**<0.001**	127.2	**<0.001**	6.3	**0.012**
80%	88	**<0.001**	63.1	**<0.001**	131.9	**<0.001**	5.7	**0.017**
90%	90.6	**<0.001**	13.5	**<0.001**	125.2	**<0.001**	7.7	**0.005**
(ii) *Random vs*. *Stratified*								
	*F*	*p*	*F*	*p*	*F*	*p*	*F*	*p*
10%	5973.5	**<0.001**	1126.6	**<0.001**	3372.9	**<0.001**	1153.5	**<0.001**
20%	5461.5	**<0.001**	1003	**<0.001**	4446.4	**<0.001**	1165.2	**<0.001**
30%	4750.8	**<0.001**	1173.4	**<0.001**	4638.9	**<0.001**	1104.1	**<0.001**
40%	6351.5	**<0.001**	1448.6	**<0.001**	3787.8	**<0.001**	1063.2	**<0.001**
50%	5673.9	**<0.001**	1643.7	**<0.001**	4917.5	**<0.001**	1051.1	**<0.001**
60%	5148.8	**<0.001**	1359.8	**<0.001**	4855.2	**<0.001**	1094	**<0.001**
70%	6120.6	**<0.001**	1328.1	**<0.001**	4410.8	**<0.001**	968.4	**<0.001**
80%	5775.6	**<0.001**	1771.6	**<0.001**	4977.8	**<0.001**	1106.4	**<0.001**
90%	5491.8	**<0.001**	874.3	**<0.001**	4711	**<0.001**	1211	**<0.001**

### Power to detect population change

**Fig 4:**
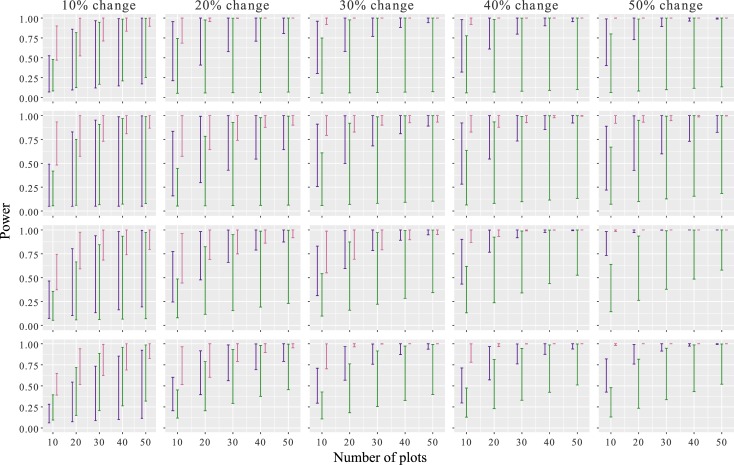
The statistical power (95% confidence intervals) to detect simulated population change of Manx shearwaters across three different scenarios in four study colonies. Simulation 1 (purple) is where population change occurred in all plots, not specific to any factor such as density or habitat, and monitoring plots were randomly selected. In simulation 2 (green), changes were simulated in a density dependent manner, simulating for example catastrophic causes of failure within colonies (e.g. disease or habitat loss) and monitoring plots were randomly selected. Simulation 3 (pink), changes were not specific to any factor such as density or habitat; however, monitoring plots were subjectively placed in the most densely-burrowed areas (upper 25%).

In simulation 1, where the population change occurred across all plots and monitoring plots were randomly selected, statistical power changed with sampling effort in a similar way across the four study sites (Fig 4). Ability to detect changes in the population was high (above 0.8) only when >20 plots were sampled, and the change was as large as 30–50%. The statistical power to detect a 30% change, for example, requires at least 30 plots to be sampled to ensure a high degree of confidence in the statistical power to detect the change. The confidence in these power estimates increased substantially with the number of plots sampled when population changes of 20% or more were simulated. However, power to detect a 10% change in the population requires considerably greater sampling effort as confidence intervals remain large at 50 plots; this was true across all sites.

In simulation 2, where the simulated changes occurred in a density dependent manner and where monitoring plots potentially came from all plots, the ability to detect population changes with a high degree of confidence was lower across all sites than in simulation 1 (Fig 4). Similarly, we see a slight increase in statistical power with increased sampling effort, yet the 95% confidence intervals remain large across all sampling efforts.

Simulation 3 shows the statistical power associated with subjectively placing monitoring plots within the most-densely burrowed areas and a simulated change across all plots identical to that of simulation 1. The ability to detect a population change was significantly improved compared to simulations 1 and 2, this was true across all sites (Fig 4). Here we demonstrate that fewer plots, located in the top 25% of densely burrowed areas, attained higher statistical power with a high degree of confidence. Similar trends were observed across all sites for each of the scenarios. Variations in the statistical power across sites within each scenario are a result of differences in the effect size, Cohen’s *d*, produced by the variance and density of breeding birds within the sampling plots.

## Discussion

We outline the uncertainty around extrapolated population estimates using three different sampling strategies on empirical data, demonstrating the clear advantage of the use of cluster and stratified sampling over random sampling approaches. Our findings illustrate that many current monitoring efforts are likely failing to detect changes in population densities as the random selection of monitoring plots reduces our ability to definitively detect change. Finally, our findings suggest that monitoring efforts should be adapted to focus on areas where there is a high density of breeding birds and little variation in plot to plot density to ensure there is high statistical power to detect change.

### Subsampling for abundance estimates

We heuristically treated the samples in our analyses as if it were the entire island. Increased subsampling effort across all sampling strategies reduced variation in, and thus increased our confidence in, abundance estimates. This emphasises that relying on low sampling efforts increases uncertainty around population estimates [44,55]. The clustering approach reduces this uncertainty and can be applied where preliminary scoping work has been carried out to determine the presence or absence of breeding individuals across all potential plots. The effectiveness of this clustering approach, however, is determined by the distribution of the breeding population. For example, many of the plots sampled on High Island contained no breeding Manx shearwaters and clustering had a large impact, whereas most plots on Skomer contained at least one breeding pair of Manx shearwaters and clustering had little effect (Fig 3). Thus, cluster sampling is most effective when the population is aggregated and patchily distributed.

On the other hand, stratification dramatically increased confidence in the estimates for all colonies. Two points are relevant with respect to the approach we took and its general applicability. First, typically in ecology, stratified sampling is not multi-stage stratification, and sampling is typically carried out in defined strata across geographical space (e.g. habitat fragments, distance from the coast). However, the approach we took is likely a reflection of habitat type, as the patchy distribution of burrow-nesting species is largely determined by the quality and availability of suitable breeding habitat [56,57]. Second, multi-stage stratification based on density is only possible where previous whole-island efforts have been carried out to establish the distribution and density of the population. We suggest this approach is valid for repeat censuses in species, such as the Manx shearwater, that show high breeding site fidelity from year to year. The general applicability of this approach, however, should be limited to species where habitat-based stratifications are discernible (e.g. [57,58]), and where habitat changes that could alter the distribution of breeding areas are readily observed through habitat assessments. Although some of the most obvious examples of such species come from avian groups (seabirds, waterbirds), in principle this should apply across all animal taxa where site fidelity is the norm [12,59], and indeed across all perennial plants [60].

Much of the literature on seabird census methods outlines that the increased complexity of the study design required to obtain reliable abundance estimates is associated with higher costs [6,61]. Our results show that low sampling efforts carried out in a random manner are unlikely to generate reliable abundance estimates. However, the difference between the random approach and the stratified approach clearly favours a stratified method. Further work is needed to understand the most efficient and realistic way of stratifying sample plots. This has been briefly discussed in Perrins *et al*. [44], where they demonstrated that apportioning sampling plots into two groups, coastal and inland areas, was effective on Skomer. However, the effectiveness of this simple clustering is likely to vary across sites and further work is required to identify the habitat and topographical features that determine the distribution of burrows. These have been explored in other burrow-nesting seabird species [57,58], but to date no study has looked at this for Manx shearwaters.

### Power analyses

In much of the literature, statistical power is examined over a time series, reporting high power to detect low annual percentage changes in population size (ca. 1% -10%) over periods of typically 5–50 years (e.g. [24,62]). These studies are largely focused on breeding populations in which counts of all individuals are attainable across years, with the aim of estimating the duration of study required to detect specific annual rates of change. However, national censuses, and for many burrow-nesting seabird species, even colony censuses, typically occur much less frequently. In Britain and Ireland, national censuses of seabirds occur every 10–15 years and few intensive monitoring programs are in place. Thus, conclusions on breeding population trends are drawn from very few data points separated by a long period of time [42,46]. Similarly, monitoring efforts after a specific event such as habitat loss or the introduction of invasive predators may necessitate comparing, and drawing conclusions from, two data points. These attempts to quantify population level change from randomly selected plots have previously failed to produce any meaningful conclusions [63]. The power analyses reported here indicated, with random sampling, the ability to detect changes in density across two years is hindered by the variation in plot densities. This was especially true in simulation 2, where the random selection of plots combined with the restriction of change to a specific area, increased the 95% confidence intervals of statistical power. Worryingly, simulation 2 may be a more realistic representation of how changes may occur within colonies [52,64] and therefore, is most illustrative of the problem associated with the random selection of monitoring plots.

Simulation 1 and 2 show that randomly selected plots, that are not representative of the density and variation in the colony as a whole, limit our ability to detect population level change. Thus, when monitoring programs use a sampling design set out to determine the overall magnitude of population change, the program’s efficacy is determined by how representative those plots are of the population as a whole [65]. Our analyses show that with a random sampling approach an enormous proportion of the colony would need to be resurveyed to account for spatial variation in density, a feat that is not logistically and economically feasible for many wildlife monitoring programmes that are limited in resources. To overcome this issue in burrow-nesting seabirds, we show that subjectively distributing monitoring plots in areas of high density increased statistical power to detect modest changes by removing the enormous, variance-inflating effect of low-density plots. Additionally, the densest plots contained the majority of breeding birds due to the patchy distribution of breeding burrows, that is likely driven by favourable breeding habitats. For High island, Inishvickillane, Little Saltee and Skomer; the top 25% of plots contained approximately 58%, 60%, 42% and 46% of the population respectively.

Our findings suggest that intense baseline survey efforts are needed to establish monitoring plots that do not vary greatly in plot to plot density to increase the statistical power to detect population change. Additionally, to ensure the sample size within the plots is high, this should be carried out in areas of high-density. In our example, by restricting monitoring to plots of higher density, the monitoring approach may sacrifice the ability to detect population expansion as some of the plots may be at carrying capacity. However, by iterating samples of 10–50 plots within the top 25% of densely occupied plots in our analyses, it is likely that plots which would support expansion are included. This limitation has been noted in monitoring across a range of taxa, such as sea turtles, where static monitoring programs fail to detect expansion in breeding sites [12]. One other restriction associated with sampling areas of highest density is that other density dependent processes that effect areas of low density could be missed. However, as demonstrated in our analyses, the effort required to detect such population level changes in distribution is beyond the scope of the resources of many conservation programmes as these are costly (see [51] as an example of full cost breakdown for one study site). One approach that can be taken to tackle these limitations, though costly, would be to establish discrete monitoring efforts in areas of both low and high density that could be analysed separately.

This prioritisation of detecting decline is outlined in other seabird studies [24,66] with the recommendation that whole-island surveys are carried out at least every 5 years to ensure (i) expansion is detected and (ii) monitoring plots are objectively placed according to the colony’s distribution. These conclusions are not solely pertinent to tape-playback efforts on burrow-nesting seabirds; the same conclusions apply to other species and methods where the variation in density of monitoring plots will largely determine the power to detect population changes. To adhere to the conclusions of the analyses carried out here, considerable effort is required to obtain baseline estimates of the population with a high level of confidence when surveying colonies such as Skomer. Moreover, the amount of effort required to obtain both a reliable abundance estimate and to optimally select monitoring plots of high density is dependent on the size of the colony.

It has been suggested that for effective conservation, approximately 60–80% of a species baseline population should be maintained, making accurate baseline population estimates of great importance [62,67]. Furthermore, the variation around abundance estimates must be sufficiently small to detect an acceptable change in population density over time. Our study suggests that random selection of monitoring plots, irrespective of colony size and distribution, will likely fail to detect modest population changes due to the enormous influence of plots that vary in density. Additionally, to reduce other potential sources of error, across a species range a common set of methods should be established that (a) are simple in execution and (b) use sampling approaches with consideration of the key issues raised in this paper. Creating standardized approaches will produce comparable datasets that can be used to assess the impact of future perturbations, including resource patch use and climate scenarios on populations at large scales.

## Supporting information

S1 File Census data required to repeat the analyses performed here. (CSV)
